# Current Status of the Liquid-Metal-Jet X-ray Source Technology

**DOI:** 10.1063/4.0000869

**Published:** 2025-10-27

**Authors:** Camilla Storaa, Björn A. M. Hansson, Mikael Otendal, Martin Norrefeldt

**Affiliations:** Excillum, Kista, MN, Sweden

## Abstract

High-end x-ray diffraction and scattering techniques such as high-resolution XRD, protein crystallography, and SAXS rely heavily on the x-ray source brightness for resolution and exposure time. Traditional solid or rotating anode x-ray tubes are typically limited in brightness by when the e-beam power density melts the anode. The liquid-metal-jet technology has overcome this limitation by using an anode that is already in the molten state.

We have delivered product performance of metal-jet anode x-ray sources with unprecedented brightness in the range of one order of magnitude above current state-of-the art sources. The technology has now further been developed in terms of output and reliability, using new solutions building on a decade of experiences.

This presentation will review the current status of the technology specifically in terms of stability, lifetime, flux and brightness. It will also discuss details of the liquid-metal-jet technology with a focus on the fundamental limitations of the technology. It will furthermore refer to some recent data from applications within x-ray diffraction and SAXS.

